# Higher light at night exposure is associated with increased risk of diabetes mellitus: A cross-sectional study of the NHANES database

**DOI:** 10.1097/MD.0000000000048904

**Published:** 2026-05-22

**Authors:** Lu He, Cheng Zeng, Cong Liu, Ying Li, Ren Lin, Jing Hu, Rong Hu, Liqing Wei, LiJuan Xu, Changzheng Chen

**Affiliations:** a Health Management Center, Renmin Hospital of Wuhan University, Wuhan, Hubei, China; b Department of Joint Surgery, Wuhan Orthopaedics Hospital of Integrated Traditional Chinese and Western Medicine, Wuhan, China; c Wuhan Wuchang Hospital, Wuhan University of Science and Technology, Wuhan, China; d Eye Center, Renmin Hospital of Wuhan University, Wuhan, Hubei, China.

**Keywords:** diabetes mellitus, light at night, metabolic health

## Abstract

While evidence links light at night (LAN) to metabolic disturbances, large-scale, population-based studies of objectively-measured LAN exposure remain limited. This study investigates the association between LAN and the risk of diabetes mellitus (DM) among US adults using data from the National Health and Nutrition Examination Survey. Cross-sectional data from the 2011 to 2014 National Health and Nutrition Examination Survey cycles were analyzed, focusing on participants with complete data on both LAN and DM. Participants were stratified by LAN level. Multivariable logistic regression analyses were employed to examine the association between LAN and DM. Multivariable linear regression analyses assessed relationships between LAN and diabetes risk markers, including hemoglobin A1c (HbA1c), fasting blood glucose, fasting serum insulin, and Homeostasis Model Assessment of Insulin Resistance (HOMA-IR). Subgroup and sensitivity analyses with multiple imputation tested robustness. A total of 4498 participants were enrolled. The prevalence of diabetes was higher in the high-LAN exposure group compared to the no-LAN group (21.01% vs 14.48%). Consistent with this, in the fully adjusted model, high-LAN exposure was independently associated with a significantly increased risk of DM (odds ratio [OR] = 1.39, 95% confidence interval [CI]: 1.00–1.92). High-LAN exposure was linked to elevated HbA1c and fasting blood glucose in initial models. However, these associations weakened and became nonsignificant after full adjustment for covariates. No significant associations were observed for fasting serum insulin or HOMA-IR. Subgroup analyses showed the association between LAN and DM was more pronounced in middle-aged adults (40–64 years), individuals with obesity, and those with dyslipidemia or hypertension. However, no significant effect modification was found across subgroups (all *P* for interaction > .05). Sensitivity analyses confirmed the robustness of these findings. This study uses objective actigraphy-based LAN measurement and shows its independent association with DM, especially in metabolically susceptible subgroups. These findings identify LAN as a modifiable environmental risk factor and support its relevance to DM prevention and public health strategy.

## 1. Introduction

Throughout evolutionary history, the biological systems of living organisms have synchronized with the Earth’s 24-hour rotation, giving rise to endogenous circadian rhythms that regulate a wide array of physiological, behavioral, and metabolic processes. In humans, this temporal organization is tightly entrained to the natural light-dark cycle, with light serving as the dominant zeitgeber (time-giver) that coordinates the central circadian clock located in the suprachiasmatic nucleus (SCN) of the hypothalamus.^[1]^

Artificial lighting, initiated by the Industrial Revolution and further amplified by electric lighting, television, computers, and smartphones, has greatly extended the hours of exposure to light. A satellite assessment found that about 83% of the world’s population lives with light pollution, especially in cities and industrial regions.^[2]^ From 2012 to 2016, the Earth’s artificially illuminated outdoor area increased by 2.2% per year, with total emissivity rising by 1.8% annually and the same rate of increase for continuously brightened areas.^[3]^

This widespread exposure to artificial light disrupts the natural light-dark cycle, altering sleep-wake patterns and contributing to various health problems, including circadian rhythm disorders, cognitive decline, mood disturbances, metabolic dysregulation, increased cancer risk, and ocular conditions such as cataracts and vision deterioration.^[4]^ Of particular concern is the growing body of evidence linking light at night (LAN) exposure to metabolic dysregulation, notably elevated risk of diabetes mellitus (DM).^[5-16]^

Despite increasing recognition of LAN as a metabolic disruptor, the existing literature predominantly relies on estimates of ambient light exposure derived from satellite data or interactive maps.^[17]^ While informative, these methods do not adequately reflect personal light exposure within indoor environments, where individuals typically spend over 80% of their time. Moreover, existing epidemiological studies on indoor LAN exposure remain limited by modest sample sizes, and often rely on portable light meters,^[5-8]^ actigraphy,^[9]^ or controlled experimental conditions with specified light intensities.^[10-13]^ These studies are further restricted by narrow demographic representation, focusing largely on elderly populations aged 60 or older^[5,6,9]^ or on young, healthy adults in their 20s.^[7,10-13]^ Given that the body’s sensitivity to light exposure and melatonin suppression diminishes with age,^[18,19]^ studies with broader age representation are essential.

Therefore, we conducted a cross-sectional study based on the NHANES database to explore the association between LAN and DM in the general US population, providing critical insights into the field of environmental health.

## 2. Materials and methods

### 2.1. Survey description

The National Health and Nutrition Examination Survey (NHANES) is a cross-sectional survey. It is conducted by the National Center for Health Statistics (NCHS). All NHANES study protocols were reviewed and approved by the NCHS Research Ethics Review Board. Written informed consent was obtained from all participants. Detailed study designs and data are publicly available at www.cdc.gov/nchs/nhanes. NHANES staff first interviewed participants in their homes to collect demographic and health-related information. Later, participants underwent physical examinations and laboratory tests at mobile examination centers. This analysis used NHANES data from the 2011–2012 to 2013–2014 cycles, accessed on February 14, 2025. These cycles were selected because the physical activity monitor component, providing objective measures of light exposure, was only available during this period. All data were de-identified, and no participant identifiers were available to the authors.

### 2.2. Study population

**Figure 1.**
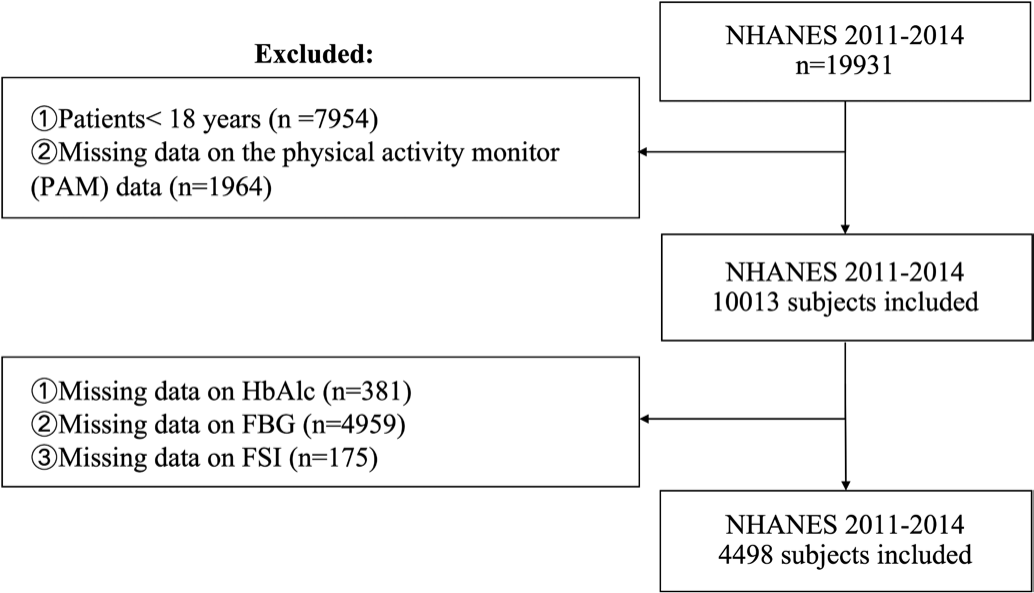
Flow chart of participants selection from the NHANES 2011 to 2014. FBG = fasting blood glucose, FSI = fasting serum insulin, HbA1c = hemoglobin A1c, NHANES = National Health and Nutrition Examination Survey, PAM = physical activity monitor.

A total of 19,931 participants from NHANES 2011 to 2014 were included in this study. Exclusions were made for participants under 18 years of age (n = 7954), those missing light or activity data (n = 1964), and those with incomplete data for glycosylated hemoglobin (HbA1c, n = 381), fasting blood glucose (FBG, n = 4959), or fasting serum insulin (FSI, n = 175). The final sample comprised 4498 individuals (Fig. 1).

### 2.3. Exposure variable

Activity and light data were collected using wrist-worn GT3X + ActiGraph devices (ActiGraph, Pensacola), which measure triaxial acceleration and ambient light levels. Participants were instructed to wear the physical activity monitor on their nondominant wrist day and night for 7 days, starting on the day of the mobile examination centers exam. The device recorded triaxial acceleration at 80 Hz and ambient light levels at 1 Hz. These data were processed to derive perminute measurements of light (in lux) and activity, quantified using the monitor-independent movement summary index, which summarizes physical activity levels. NHANES classified each 1-minute as either wake, sleep, or nonwear time using an open-source algorithm. The algorithm assigned a confidence value between 0.0 and 1.0 to indicate its certainty in classifying each period as wake, sleep, or nonwear. When classification was not possible, periods were marked as “unknown.” Further details on the algorithm are available on the NHANES website.^[20]^

White light data (measured in lux) from the ActiGraph light sensor were used to calculate LAN. LAN was defined as the averaged light exposure within the 5-hour activity nadir (L5), a proxy for rest. The L5 LAN variable was derived using R version 4.1. Actigraphy data from the 2011–2012 to 2013–2014 NHANES cycles were trimmed to exclude incomplete measurements on the first and ninth days, leaving data from the 7 consecutive full days (days 2–8). Triaxial accelerometry data were used to determine the L5 start time each day, from which LAN was derived as the average white light exposure across the L5 period. Participants were subsequently categorized into 3 groups based on the median LAN exposure (0.84 lux): none (0 lux), low (0–0.84 lux), and high (> 0.84 lux).

### 2.4. Outcome variables

The outcome variables included the prevalence of DM, and risk markers for DM, including FBG (mmol/L), HbA1c (%), FSI (pmol/L), and homeostasis model assessment of insulin resistance (HOMA-IR).

DM was diagnosed based on a comprehensive questionnaire and laboratory test results, with criteria including any of the following: FBG ≥ 7.0 mmol/L (126 mg/dL), oral glucose tolerance test 2-hour plasma glucose (PG2H) ≥ 11.1 mmol/L (200 mg/dL), HbA1c ≥ 6.5%, self-reported diabetes, or current use of hypoglycemic therapy.^[21]^ Homeostasis model assessment of insulin resistance (HOMA-IR) was calculated using the formula: [FBG (mmol/L)*FSI (μU/mL)]/22.5.^[22]^ FSI (pmol/L) was measured, and in the HOMA-IR formula, it was converted to μU/mL.

### 2.5. Covariates

Covariates for this study were selected based on the following criteria: demographic data, variables associated with DM in previous studies, and other variables based on clinical experience. The selected covariates included: age (years), gender, race, education level, marital status, smoking status, alcohol consumption, systolic blood pressure (SBP, mm Hg), body mass index (BMI, kg/m^2^), sleep duration, diet quality, and total daily electronic screen time.

Race/ethnicity categories included: Mexican American, Other Hispanic, Non-Hispanic White, non-Hispanic Black, and other race. Education level was grouped as less than high school, high school or equivalent, and college or above. Marital status was categorized as married/living with a partner, widowed/divorced/separated, or never married. Smoking status was categorized into current smokers, never smokers, and former smokers. Alcohol consumption was classified as: nondrinker, 1 to <5 drinks per month, 5 to <10 drinks per month, or 10+ drinks per month. The poverty income ratio (PIR) was used to categorize household income into low (PIR ≤ 1.3), medium (PIR > 1.3 to 3.5), and high (PIR > 3.5). Physical activity (PA) was self-reported using the Global Physical Activity Questionnaire, classifying participants as inactive (<600 MET min/wk) or active (≥600 MET min/wk, equivalent to 150 min/wk of moderate-intensity or 75 min/wk of vigorous-intensity PA).^[23]^

Sleep duration was self-reported by the question “How much sleep do you usually get at night on weekdays or workdays?” Diet quality was assessed using Healthy Eating Index-2020 (HEI-2020) scores, calculated from 2 24-hour dietary recall interviews. Higher HEI-2020 scores (range 0–100) indicate better adherence to the Dietary Guidelines for Americans.^[24]^ Total daily electronic screen time was derived by summing self-reported hours spent watching TV/videos and using a computer, as captured by the physical activity questionnaire.^[25]^ Screen time was defined as low (<2 h/d), moderate (2–5 h/d), or high (≥5 h/d).

### 2.6. Statistical analyses

#### 2.6.1. Data presentation and group comparisons

LAN levels were divided into 3 groups: no LAN, low LAN, and high LAN. Continuous variables were summarized using mean ± standard deviation or median (IQR), depending on the data distribution. Differences between continuous variables were assessed using one-way ANOVA for normally distributed data, or the Kruskal-Wallis test for non-normally distributed data. Categorical variables were described as counts and percentages. Differences between categorical variables were analyzed using the Chi-square test or Fisher’s exact test.

#### 2.6.2. Missing data handling

The proportions of missing data for each covariate were as follows: education levels (5.07%, n = 228), PIR (7.38%, n = 332), smoking status (2.25%, n = 101), alcohol user (7.36%, n = 331), sleep duration (0.16%, n = 7), HEI-2020 (13.98%, n = 629), BMI (0.87%, n = 39), and SBP (1.73%, n = 78). Missing data in categorical covariates were addressed by creating a distinct “Missing” category, which was included in the regression models. For continuous covariates with missing values, imputation was performed using the sample median. All primary analyses were conducted on this dataset. To evaluate the robustness of our findings, we performed a sensitivity analysis using multiple imputation. Specifically, we used the MI procedure in R, generating 5 imputed datasets via a chained equation approach to account for uncertainty in the missing values.

#### 2.6.3. Regression models

Three models were used for statistical analysis: model 1 (univariate logistic regression), model 2 (adjusted for age, gender, and race), and model 3 (additionally adjusted for education level, PIR, smoking, alcohol use, PA, SBP, BMI, sleep duration, HEI-2020, and screen time). Multivariate linear regression was used to analyze the associations of LAN with DM risk markers (FBG, HbA1c, FSI, and HOMA-IR). Multivariate logistic regression models were employed to assess the relationship between LAN and DM, with odds ratios (ORs) and 95% confidence intervals (CIs) calculated for each model.

#### 2.6.4. Subgroup analyses

Subgroup analysis was performed to examine potential interactions and control for confounding variables, including gender, age, race, education level, PIR, smoking status, alcohol use, PA, general obesity, hypertension, dyslipidemia, sleep duration, HEI-2020, and screen time. Participants were classified into 3 age groups: 18 to 39 years, 40 to 64 years, and 65+ years. Obesity classification followed World Health Organization standards: normal weight (18.5 kg/m^2^ ≤ BMI < 25 kg/m^2^), overweight (25 kg/m^2^ ≤ BMI < 30 kg/m^2^), and obese (BMI ≥ 30 kg/m^2^). Hypertension was defined as SBP ≥ 140 mm Hg and/or DBP ≥ 90 mm Hg, or self-reported hypertension with use of antihypertensive medication. Dyslipidemia was defined as total cholesterol ≥ 5.2 mmol/L, low-density lipoprotein cholesterol (LDL-C) ≥ 3.4 mmol/L, high-density lipoprotein cholesterol (HDL-C) < 1.0 mmol/L, triglycerides (TG) ≥ 1.7 mmol/L, or self-reported hypercholesterolemia and lipid-lowering medication use. Sleep duration was grouped as <7 h per night and ≥7 h per night. Diet quality was categorized as low (HEI-2020 score < 50) or high (HEI-2020 score ≥ 50). Multivariate logistic regression was used for subgroup analysis. If the interaction *P* value was nonsignificant, it indicated no evidence of effect modification; otherwise, a special population effect may have occurred.

#### 2.6.5. Model assumptions validation

A systematic validation of key assumptions of the linear regression model was performed. The Durbin–Watson statistic confirmed the independence of residuals. Homoscedasticity was verified by examining scatter plots of standardized residuals versus predicted values, while normality of residuals was assessed using histograms with overlaid normal curves and Q–Q plots. Variance inflation factor (VIF) values indicated multicollinearity. In summary, all key assumptions were satisfied, supporting the appropriateness of the model specification.

#### 2.6.6. Software and significance

All statistical analyses were performed using EmpowerStats (version 4.1, www.empowerstats.com) and R (version 4.4.1, http://www.R-project.org), with statistical significance defined as *P* < .05.

## 3. Results

### 3.1. Characteristics of the study population

Table 1 presents the baseline characteristics of participants stratified by LAN levels. Compared to the non-LAN group, individuals in the high-LAN group were more likely to be male (52.07% vs 41.86%), non-Hispanic Black (23.81% vs 12.22%), and current smokers (21.09% vs 10.18%; all *P* < .001). From a socioeconomic perspective, high-LAN participants were more likely to belong to the low-income group (PIR ≤ 1.3: 33.51% vs 25.11%; *P* < .001). The high-LAN group demonstrated significantly shorter sleep duration (6.77 ± 1.45 vs 7.33 ± 1.34 hours; *P* < .001), an elevated prevalence of short sleep (<7 hours: 41.48% vs 23.53%; *P* < .001), lower HEI-2020 scores (51.56 ± 11.25 vs 53.65 ± 11.48; *P* = .002), and a higher proportion with low diet quality (HEI < 50: 44.68% vs 30.32%; *P* < .001). Clinically, this group also exhibited a higher prevalence of obesity (BMI ≥ 30 kg/m^2^: 37.29% vs 31.45%), hypertension (43.84% vs 37.33%), and diabetes (21.01% vs 14.48%). In terms of continuous variables, they presented elevated BMI (29.16 ± 7.07 vs 28.16 ± 6.45 kg/m^2^), SBP (122.76 ± 17.79 vs 120.39 ± 16.26 mm Hg), and HbA1c levels (5.80 ± 1.17% vs 5.63 ± 0.86%).

**Table 1:** Baseline characteristics among study population stratified by LAN, NHANES, 2011 to 2014 (N = 4498).

Variables	Overall	Non-LAN 0 lux	Low LAN (0–0.84 lux)	High LAN (>0.84 lux)	*P*-value
n	4498	442	1809	2247	
Age (yr)	47.70 ± 18.28	48.48 ± 18.67	46.51 ± 18.50	48.51 ± 17.98	**.002**
Age, n (%)					**<.001**
18–39 yr	1654 (36.77)	148 (33.48)	731 (40.41)	775 (34.49)	
40–64 yr	1876 (41.71)	187 (42.31)	701 (38.75)	988 (43.97)	
65+ yr	968 (21.52)	107 (24.21)	377 (20.84)	484 (21.54)	
Gender, n (%)					**<.001**
Male	2186 (48.60)	185 (41.86)	831 (45.94)	1170 (52.07)	
Female	2312 (51.40)	257 (58.14)	978 (54.06)	1077 (47.93)	
Race, n (%)					**<.001**
Mexican American	532 (11.83)	52 (11.76)	229 (12.66)	251 (11.17)	
Other Hispanic	448 (9.96)	51 (11.54)	190 (10.50)	207 (9.21)	
Non-Hispanic White	1898 (42.20)	211 (47.74)	764 (42.23)	923 (41.08)	
Non-Hispanic Black	954 (21.21)	54 (12.22)	365 (20.18)	535 (23.81)	
Other Race	666 (14.81)	74 (16.74)	261 (14.43)	331 (14.73)	
Education levels, n (%)					.184
Less than high school	951 (21.14)	86 (19.46)	379 ((20.95)	486 (21.63)	
High school or equivalent	905 (20.12)	77 (17.42)	365 (20.18)	463 (20.61)	
College or above	2414 (53.67)	255 (57.69)	959 (53.01)	1200 (53.40)	
Missing	228 (5.07)	24 (5.43)	106 (5.86)	98 (4.36)	
PIR, n (%)					**<.001**
Low	1495 (33.24)	111 (25.11)	631 (34.88)	753 (33.51)	
Middle	1451 (32.26)	150 (33.94)	564 (31.18)	737 (32.80)	
High	1220 (27.12)	146 (33.03)	452 (24.99)	622 (27.68)	
Missing	332 (7.38)	35 (7.92)	162 (8.96)	135 (6.01)	
Smoking status, n (%)					**<.001**
Never	2531 (56.27)	269 (60.86)	1037 (57.32)	1225 (54.52)	
Former	1004 (22.32)	118 (26.70)	389 (21.50)	497 (22.12)	
Current	862 (19.16)	45 ((10.18)	343 (18.96)	474 (21.09)	
Missing	101 (2.25)	10 (2.26)	40 (2.21)	51 (2.27)	
Alcohol user, n (%)					.077
Nondrinker	1178 (26.19)	112 (25.34)	489 (27.03)	577 (25.68)	
1–5 drinks/mo	2123 (47.20)	208 (47.06)	858 (47.43)	1057 (47.04)	
5–10 drinks/mo	291 (6.47)	24 (5.43)	104 (5.75)	163 (7.25)	
10+ drinks/mo	575 (12.78)	59 ((13.35)	210 (11.61)	306 (13.62)	
Missing	331 (7.36)	39 (8.82)	148 (8.18)	144 (6.41)	
PA, n (%)					.701
Not active	1721 (38.26)	172 (38.91)	703 (38.86)	846 (37.65)	
Active	2777 (61.74)	270 (61.09)	1106 (61.14)	1401 (62.35)	
Sleep duration (hours)	6.90 ± 1.41	7.33 ± 1.34	6.94 ± 1.37	6.77 ± 1.45	**<.001**
Sleep duration group, n(%)					**<.001**
<7 h	1679 (37.33)	104 (23.53)	643 (35.54)	932 (41.48)	
≥7 h	2812 (62.52)	336 (76.02)	1164 (64.34)	1312 (58.39)	
Missing	7 (0.16)	2 (0.45)	2 (0.11)	3 (0.13)	
HEI-2020	51.87 ± 11.31	53.65 ± 11.48	51.83 ± 11.31	51.56 ± 11.25	**.002**
HEI-2020 group, n(%)					**<.001**
<50	1934 (43.00)	134 (30.32)	796 (44.00)	1004 (44.68)	
≥50	1935 (43.02)	222 (50.23)	766 (42.34)	947 (42.15)	
Missing	629 (13.98)	86 (19.46)	247 (13.65)	296 (13.17)	
Acreen time, n(%)					.216
<2 h/d	881 (19.59%)	94 (21.27)	349 (19.29)	438 (19.49)	
2–5 h/d	2166 (48.15%)	217 (49.10)	898 (49.64)	1051 (46.77)	
≥5 h/d	1451 (32.26%)	131 (29.64)	562 (31.07)	758 (33.73)	
BMI (kg/m^2^)	28.83 ± 6.97	28.16 ± 6.45	28.58 ± 6.95	29.16 ± 7.07	**.003**
BMI group, n(%)					.060
<25.0	1419 (31.55)	148 (33.48)	608 (33.61)	663 (29.51)	
25.0–30.0	1428 (31.75)	151 (34.16)	552 (30.51)	725 (32.27)	
≥30.0	1612 (35.84)	139 (31.45)	635 35.10)	838 (37.29)	
Missing	39 (0.87)	4 (0.90)	14 (0.77)	21 (0.93)	
SBP (mm Hg)	122.02 ± 17.46	120.39 ± 16.26	121.50 ± 17.29	122.76 ± 17.79	**.008**
DBP (mm Hg)	68.86 ± 12.61	68.46 ± 11.38	68.92 ± 12.43	68.88 ± 12.99	.782
Hypertension, n (%)					**<.001**
No	2595 (57.69)	270 (61.09)	1098 (60.70)	1227 (54.61)	
Yes	1825 (40.57)	165 (37.33)	675 (37.31)	985 (43.84)	
Missing	78 (1.73)	7 (1.58)	36 (1.99)	35 (1.56)	
Dyslipidemia, n (%)					.879
No	1516 (33.70)	146 (33.03)	617 (34.11)	753 (33.51)	
Yes	2982 (66.30)	296 (66.97)	1192 (65.89)	1494 (66.49)	
Diabetes, n (%)					**.002**
No	3632 (80.75)	378 (85.52)	1479 (81.76)	1775 (78.99)	
Yes	866 (19.25)	64 (14.48)	330 (18.24)	472 (21.01)	
HOMA-IR	3.97 ± 7.68	3.76 ± 5.32	3.96 ± 8.85	4.03 ± 7.30	.789
Laboratory data					
HBA1C (%)	5.74 ± 1.09	5.63 ± 0.86	5.70 ± 1.04	5.80 ± 1.17	**.001**
FBG (mmol/L)	5.94 ± 1.86	5.75 ± 1.56	5.92 ± 1.82	5.99 ± 1.95	.050
FSI (pmol/L)	82.62 ± 116.51	82.01 ± 78.39	81.71 ± 122.10	83.48 ± 118.20	.884

Mean ± SD for continuous variables, *P*-values was calculated by one-way ANOVA or Kruskal–Wallis test.

% for categorical variables, *P*-values was calculated by the Chi-square test or Fisher’s exact test.

Bold values indicate statistical significance (*P* < .05).

BMI = body mass index, DBP = diastolic blood pressure, FBG = fasting blood glucose, FSI = fasting serum insulin, HBA1c = hemoglobin A1c, HEI-2020 = healthy eating index-2020, HOMA-IR = homeostasis model assessment of insulin resistance, PA = physical activity, PIR = poverty-to-income ratio, SBP = systolic blood pressure.

### 3.2. Association between LAN and DM risk

Table 2 summarizes the associations between LAN and the odds of DM. In the unadjusted model (model 1), high-LAN exposure was associated with a significantly increased risk of DM compared to the non-LAN group (OR = 1.57, 95% confidence interval [CI]: 1.18–2.09, *P* = .002), with a significant dose-response trend (*P* for trend < .001). After adjusting for age, sex, and race (model 2), this association persisted for both low-LAN (OR = 1.43, 95% CI: 1.05–1.96, *P* = .024) and high-LAN (OR = 1.57, 95% CI: 1.16–2.13, *P* = .004), with a maintained trend (*P* for trend = 0.007). In the fully adjusted model (model 3), which additionally controlled for socioeconomic status (education level, PIR), lifestyle behaviors (smoking, alcohol use, physical activity, sleep duration, HEI-2020 and screen time), and clinical parameters (SBP and BMI), the association remained statistically significant for the high-LAN group (OR = 1.39, 95% CI: 1.00–1.92, *P* = .048).

**Table 2:** The associations between LAN and prediabetes or diabetes.

	Model 1		Model 2		Model 3	
	β (95%CI)/OR (95%CI)	*P* value	β (95%CI)/OR (95%CI)	*P* value	β (95%CI)/OR (95%CI)	*P* value
Diabetes						
LAN group						
Non-LAN	Reference		Reference		Reference	
Low LAN	1.32 (0.99, 1.76)	.062	1.43 (1.05, 1.96)	**.024**	1.31 (0.94, 1.82)	.112
High LAN	1.57 (1.18, 2.09)	**.002**	1.57 (1.16, 2.13)	**.004**	1.39 (1.00, 1.92)	**.048**
* P* for trend	**<.001**		**.007**		.077	

Model 1 adjust for None.

Adjust model 2 adjust for age, gender, race.

Adjust model 3 adjust for age, gender, race, education level, PIR, smoking, alcohol use, PA, SBP, BMI, sleep duration, HEI-2020 and screen time.

Bold values indicate statistical significance (*P* < .05).

BMI = body mass index, CI = confidence interval, HEI-202 = healthy eating index-2020, OR = odds ratio, PA = physical activity, PIR = poverty-to-income ratio, SBP = systolic blood pressure.

### 3.3. Association between LAN and risk markers of diabetes mellitus

The associations between different LAN and DM risk markers were analyzed using multivariable linear regression models (Table 3). In the unadjusted model 1, the high-LAN group was significantly associated with higher levels of HbA1c (β = 0.17, 95% CI: 0.06–0.28) and FBG (β = 0.23, 95% CI: 0.04–0.42), with significant dose-response trends (*P* for trend < .05). In model 2, these associations remained significant for the High-LAN group (HbA1c: β = 0.13, 95% CI: 0.03–0.24; FBG: β = 0.19, 95% CI: 0.01–0.37). The association between the Low-LAN group and FBG also became significant (β = 0.19, 95% CI: 0.01–0.38). However, in model 3, the associations between LAN and both HbA1c and FBG were attenuated and lost statistical significance. No significant associations were observed between LAN and FSI or HOMA-IR in any of the models.

**Table 3:** The associations between LAN and risk markers of DM.

	Model 1		Model 2		Model 3	
	β (95%CI)/OR (95%CI)	*P* value	β (95%CI)/OR (95%CI)	*P* value	β (95%CI)/OR (95%CI)	*P* value
HBA1C						
LAN group						
Non-LAN	Reference		Reference		Reference	
Low LAN	0.07 (−0.04, 0.19)	.203	0.08 (−0.03, 0.19)	.138	0.04 (−0.06, 0.15)	.421
High LAN	0.17 (0.06, 0.28)	**.002**	0.13 (0.03, 0.24)	**.014**	0.08 (−0.03, 0.18)	.143
* P* for trend	**<.001**		**.009**		.107	
FBG						
LAN group						
Non-LAN	Reference		Reference		Reference	
Low LAN	0.17 (−0.02, 0.36)	.087	0.19 (0.01, 0.38)	**.043**	0.14 (−0.05, 0.32)	.148
High LAN	0.23 (0.04, 0.42)	**.016**	0.19 (0.01, 0.37)	**.042**	0.11 (−0.07, 0.29)	.248
* P* for trend	**.021**		.147		.567	
FSI						
LAN group						
Non-LAN	Reference		Reference		Reference	
Low LAN	−0.30 (−12.42, 11.82)	.961	−1.65 (−13.77, 10.47)	.790	−4.79 (−16.20, 6.62)	.411
High LAN	1.47 (−10.41, 13.36)	.808	−0.57 (−12.50, 11.35)	.925	−6.63 (−17.90, 4.64)	.249
* P* for trend	.670		.935		.268	
HOMA-IR						
LAN group						
Non-LAN	Reference		Reference		Reference	
Low LAN	0.20 (−0.60, 1.00)	.621	0.16 (−0.64, 0.96)	.693	0.04 (−0.73, 0.80)	.922
High LAN	0.27 (−0.51, 1.05)	.497	0.14 (−0.65, 0.92)	.732	−0.08 (−0.83, 0.68)	.845
* P* for trend	.520		.842		.695	

Model 1 adjust for None.

Adjust model 2 adjust for age, gender, race.

Adjust model 3 adjust for age, gender, race, education level, PIR, smoking, alcohol use, PA, SBP, BMI, sleep duration, HEI-2020 and screen time.

Bold values indicate statistical significance (*P* < .05).

CI = confidence interval, BMI = body mass index, FBG = fasting blood glucose, FSI = fasting serum insulin, HBA1c = hemoglobin A1c, HEI-2020 = healthy eating index-2020, HOMA-IR = homeostasis model assessment of insulin resistance, OR = odds ratio, PA = physical activity, PIR = poverty-to-income ratio, SBP = systolic blood pressure.

### 3.4. Subgroup analyses

**Figure 2.**
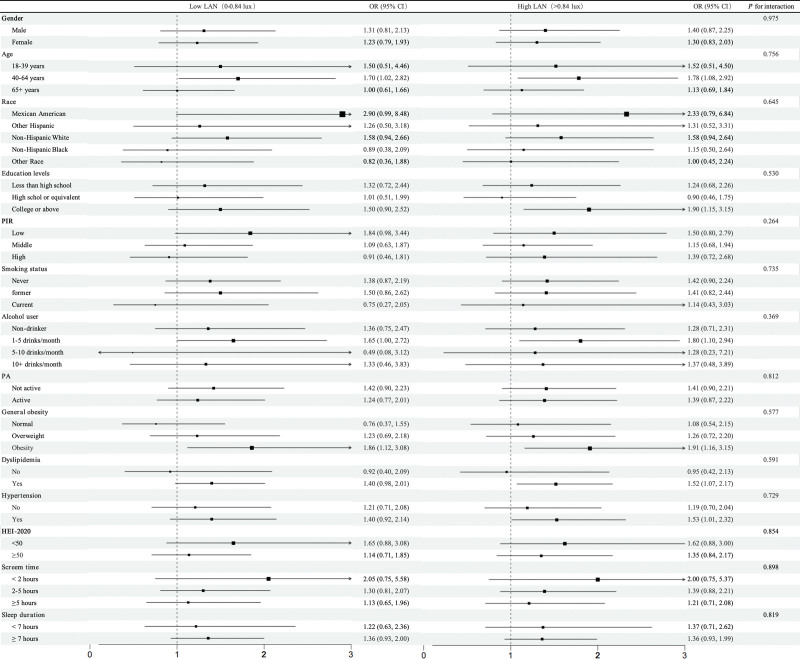
Subgroup analysis of the associations between LAN and DM. CI =confidence interval, DM = diabetes mellitus, LAN = light at night, OR = odds ratio, PA = physical activity.

Stratified analyses (Fig. 2) assessed whether the association between LAN and DM varied across subgroups. Although no statistically significant interaction effects were observed across all subgroups (all *P* for interaction > .05), significant dose-response relationships were identified in several specific populations. Among adults aged 40 to 64, both low-LAN and high-LAN exposures were associated with increased DM risk (low-LAN: odds ratio [OR] = 1.70, 95% CI: 1.02–2.82, *P* = .040; high-LAN: OR = 1.78, 95% CI: 1.08–2.92, *P* = .023). Participants with college education or above had a significantly elevated diabetes risk in the high LAN group (OR = 1.90, 95% CI: 1.15–3.15, *P* = .013). Individuals who reported drinking 1 to 5 times per month had significantly higher odds of DM in the high LAN group (OR = 1.80, 95% CI: 1.10–2.94, *P* = .019). Individuals with obesity had significantly elevated odds of DM in both LAN groups (low-LAN: OR = 1.86, 95% CI: 1.12–3.08, *P* = .017; high-LAN: OR = 1.91, 95% CI: 1.16–3.15, *P* = .011). Similarly, among participants with dyslipidemia (OR = 1.52, 95% CI: 1.07–2.17, *P* = .020) or hypertension (OR = 1.53, 95% CI: 1.01–2.32, *P* = .045), high LAN exposure was associated with elevated odds of DM. Although no significant interaction effects were detected overall, point estimates showed elevated risks in subgroups including middle-aged adults, highly educated individuals, moderate alcohol consumers, obesity, and patients with dyslipidemia or hypertension.

### 3.5. Sensitivity analysis

Sensitivity analysis with multiple imputation showed that associations between LAN and DM, as well as risk markers, remained consistent with the primary analysis after accounting for missing data, supporting the robustness of our findings (Table 4). Under model 3 (fully adjusted), the link between high LAN and DM was stable and statistically significant across all 5 imputed datasets (ORs: 1.41–1.42; all *P* < .05). Multiple imputation results matched the primary analysis: no significant associations were found between LAN exposure and HbA1c, FBG, FSI, or HOMA-IR (all *P* > .05) in model 3.

**Table 4:** Multiple imputation the associations between LAN and risk markers of DM.

	MI 1		MI 2		MI 3		MI 4		MI 5	
	β (95%CI)/OR (95%CI)	*P* value	β (95%CI)/OR (95%CI)	*P* value	β (95%CI)/OR (95%CI)	*P* value	β (95%CI)/OR (95%CI)	*P* value	β (95%CI)/OR (95%CI)	*P* value
Diabetes										
LAN group										
Non-LAN										
Low LAN	1.31 (0.95, 1.83)	.104	1.32 (0.95, 1.83)	.099	1.32 (0.95, 1.83)	.099	1.31 (0.94, 1.83)	.107	1.31 (0.95, 1.83)	.104
High LAN	1.41 (1.02, 1.95)	**.039**	1.42 (1.03, 1.96)	**.034**	1.41 (1.02, 1.95)	**.037**	1.41 (1.02, 1.94)	.040	1.41 (1.02, 1.94)	**.039**
* P* for trend	.060		.052		.059		.061		.061	
HBA1C										
LAN group										
Non-LAN										
Low LAN	0.04 (v0.06, 0.15)	.417	0.04 (−0.06, 0.15)	.418	0.04 (−0.06, 0.15)	.404	0.04 (−0.06, 0.15)	.422	0.04 (−0.06, 0.15)	.430
High LAN	0.08 (−0.02, 0.19)	.122	0.08 (−0.02, 0.19)	.121	0.08 (−0.02, 0.19)	.123	0.08 (−0.02, 0.18)	.131	0.08 (−0.02, 0.18)	.132
* P* for trend	.082		.080		.087		.091		.090	
FBG										
LAN group										
Non-LAN										
Low LAN	0.14 (−0.05, 0.32)	.147	0.14 (−0.04, 0.32)	.139	0.14 (−0.05, 0.32)	.141	0.14 (−0.05, 0.32)	.145	0.13 (−0.05, 0.32)	.151
High LAN	0.11 (−0.07, 0.29)	.236	0.11 (−0.07, 0.29)	.221	0.11 (−0.07, 0.29)	.234	0.11 (−0.07, 0.29)	.242	0.11 (−0.07, 0.29)	.248
* P* for trend	.541		.516		.547		.560		.562	
FSI										
LAN group										
Non-LAN										
Low LAN	−4.76 (−16.17, 6.64)	.413	−4.71 (−16.13, 6.70)	.418	−4.77 (−16.19, 6.65)	.413	−4.36 (−15.78, 7.05)	.454	−4.76 (−16.17, 6.65)	.414
High LAN	−6.76 (−18.01, 4.50)	.240	−6.62 (−17.89, 4.65)	.249	−6.89 (−18.17, 4.39)	.231	−6.62 (−17.89, 4.65)	.250	−6.69 (−17.96, 4.58)	.245
* P* for trend	.251		.264		.237		.242		.259	
HOMA-IR										
LAN group										
Non-LAN										
Low LAN	−0.04 (−0.81, 0.73)	.918	−0.03 (−0.80, 0.73)	.930	−0.04 (−0.81, 0.72)	.910	−0.02 (−0.79, 0.74)	.957	−0.04 (−0.80, 0.73)	.921
High LAN	−0.23 (−0.99, 0.52)	.545	−0.22 (−0.98, 0.53)	.563	−0.24 (−1.00, 0.51)	.529	−0.23 (−0.99, 0.53)	.551	−0.23 (−0.99, 0.52)	.549
* P* for trend	.385		.402		.370		.370		.389	

Adjust for age, gender, race, education level, PIR, smoking, alcohol use, PA, SBP, BMI, sleep duration, HEI-2020 and screen time.

Bold values indicate statistical significance (*P* < .05).

CI = confidence interval, BMI = body mass index, DBP = diastolic blood pressure, FBG = fasting blood glucose, FSI = fasting serum insulin, HBA1c = hemoglobin A1c, HEI-2020 = healthy eating index-2020, HOMA-IR = homeostasis model assessment of insulin resistance, OR = odds ratio, PA = physical activity, PIR = poverty-to-income ratio, SBP = systolic blood pressure.

## 4. Discussion

This study showed that objectively-measured personal exposure to LAN is associated with an increased risk of DM in US adults. In the fully adjusted model, participants in the high-LAN group had 39% higher odds of DM. The pattern of associations with glycemic markers offers crucial mechanistic insights. For example, LAN exposure was significantly associated with HbA1c and FBG in models 1 and 2. These associations weakened after comprehensive adjustment for confounders. Consistent null findings for FSI and HOMA-IR across models suggest that LAN may influence glucose metabolism through pathways independent of classic insulin resistance.

Building on prior research, our study provides a deeper understanding of the associations between LAN and DM through methodological innovation. Earlier large-scale studies depended on satellite-based measurements of outdoor LAN.^[14,15]^ The use of personal actigraphy enabled precise measurement of individual exposure. This method aligns with the prevailing view that personal monitoring yields more physiologically meaningful data than geospatial estimates in evaluating the health impacts of light.

The emerging literature on personally measured LAN provides important context for our findings. A cross-sectional study among 552 elderly adults aged 65 to 84 years found a positive association between indoor LAN and DM prevalence (OR = 1.72; 95% CI, 1.12–2.64).^[5]^ Another study in adults aged 60 and older linked LAN exposure, measured via 7-day actigraphy recordings during sleep, to an increased risk of DM (OR = 2.0; 95% CI: 1.19–3.43).^[9]^ Furthermore, a study found that an increase in LAN exposure from 17.5 lux (25th percentile) to 37.6 lux (75th percentile) was associated with a 51.2% increase in DM prevalence among the elderly.^[5]^ Obayashi et al also conducted a prospective cohort study involving 678 older adults, with a median follow-up period of 42 months. Our findings are consistent with prior research and extend the evidence to a broader adult population. Particularly noteworthy is the prospective finding from Obayashi et al, who reported that the incidence rate for diabetes was higher in the LAN group than in the dark group (incident rate ratio = 3.19).^[6]^ The UK cohort study found that brighter LAN predicted higher DM risk over nearly 8 years of follow-up (HR = 1.53).^[8]^ These longitudinal data strengthen the biological plausibility of our cross-sectional findings.

After robust adjustment for potential confounders, the associations between LAN and HbA1c or FBG were attenuated and no longer statistically significant. This pattern is consistent with a report from southern India,^[16]^ but stands in contrast to other studies documenting positive relationships.^[14]^ This discrepancy may reflect limitations of single-point metabolic measurements in cross-sectional studies. Possible confounding by glucose-lowering medications might also mask the association.

The dissociation between LAN and insulin resistance markers in our study suggests the involvement of other physiological pathways beyond classical insulin resistance. Notably, LAN was consistently associated with an increased risk of DM across all models and with elevated FBG in models 1 and 2, despite null associations with FSI or HOMA-IR. This pattern indicates that LAN may promote fasting hyperglycemia through mechanisms that do not primarily depend on peripheral insulin sensitivity. Importantly, FBG and HOMA-IR reflect distinct aspects of glucose regulation, with FBG potentially being more sensitive to hepatic glucose production and pancreatic hormone secretion.

One key pathway linking LAN to metabolic disruptions is circadian misalignment. LAN disrupts the circadian oscillator, which is driven by the CLOCK/BMAL1 complex and stabilized through PER/CRY protein feedback. This disruption alters PER1/PER2 expression, leading to circadian phase shifts.^[26]^ Experimental evidence suggests that the pancreatic islet circadian clock is particularly sensitive to LAN exposure. Disruption of islet rhythmicity can impair β-cell function and reduce glucose-stimulated insulin secretion by reducing insulin secretory pulse mass.^[27]^ In addition, through neuroendocrine pathways, LAN suppresses SCN-regulated melatonin secretion via intrinsically photosensitive retinal ganglion cells (ipRGCs) while simultaneously disrupting peripheral circadian synchronization. This dual disruption significantly contributes to metabolic dysfunction.^[28]^ Moreover, LAN can rapidly induce co-dysregulation of hepatic clock genes and gluconeogenic genes via autonomic nervous pathways, potentially contributing to changes in glucose homeostasis.^[29]^ Finally, LAN may interfere with adaptive thermogenesis in brown adipose tissue through non-SCN neural sympathetic pathways, further contributing to impaired glucose tolerance.^[30]^

The consistent pattern of heightened vulnerability observed across multiple subgroups – particularly middle-aged adults, individuals with obesity, and those with cardiometabolic comorbidities – suggests that preexisting metabolic susceptibility may amplify LAN-related diabetes risk. Although these subgroup analyses did not reach statistical significance for interaction terms, the consistent direction and magnitude of effects across these populations warrant attention in future studies with larger sample sizes.

Despite the robustness of our findings, several limitations should be noted. First, the cross-sectional design limits causal inferences, and longitudinal studies are needed to assess whether chronic LAN exposure causes diabetes. Second, wrist-worn actigraphy, though practical, may underestimate ambient light due to body coverage or user behavior, potentially leading to misclassification of exposure. Third, we could not examine light characteristics like wavelength, timing, or duration, which may influence glucose metabolism. Fourth, although we adjusted for numerous covariates, residual confounding from unmeasured factors such as genetic predisposition and shift work may persist. Finally, this analysis did not use NHANES survey weights or account for the complex survey design, so the absolute estimates and standard errors may not reflect the US population.

## 5. Conclusions

Our findings suggest that exposure to LAN increases the risk of DM, highlighting the importance of environmental factors in managing diabetes risk. As part of broader public health recommendations, reducing nighttime light exposure – such as turning off lights – could be a simple, cost-effective way to mitigate this risk.

## Acknowledgments

We extend our gratitude to all participants of the NHANES.

## Author contributions

**Conceptualization:** Ying Li, Changzheng Chen.

**Data curation:** Cheng Zeng, Cong Liu, Liqing Wei.

**Formal analysis:** Cheng Zeng, Cong Liu.

**Investigation:** Ying Li, Ren Lin, Jing Hu.

**Methodology:** Lu He, Ying Li, Ren Lin, Jing Hu.

**Project administration:** LiJuan Xu, Changzheng Chen.

**Software:** Lu He, Ying Li, Ren Lin, Jing Hu.

**Supervision:** LiJuan Xu.

**Validation:** Rong Hu.

**Visualization:** Lu He.

**Writing – original draft:** Lu He.

**Writing – review & editing:** Lu He, LiJuan Xu, Changzheng Chen.
